# DNA computing for gastric cancer analysis and functional classification

**DOI:** 10.3389/fgene.2022.1064715

**Published:** 2022-11-24

**Authors:** Congzhou Chen, Xin Chen, Xin Li, Xiaolong Shi

**Affiliations:** ^1^ School of Computer Science, Peking University, Beijing, China; ^2^ Institute of Computing Science and Technology, Guangzhou University, Guangzhou, China; ^3^ Department Genecology 2, Renmin Hospital of Wuhan University, Wuhan, China

**Keywords:** co-expression network, DNA computing, cancer diagnosis, miRNA biomarkers, DNA strand displacement (DSD)

## Abstract

Early identification of key biomarkers of malignant cancer is vital for patients’ prognosis and therapies. There is research demonstrating that microRNAs are important biomarkers for cancer analysis. In this article, we used the DNA strand displacement mechanism (DSD) to construct the DNA computing system for cancer analysis. First, gene chips were obtained through bioinformatical training. These microRNA data and clinical traits were obtained from the Cancer Genome Atlas (TCGA) dataset. Second, we analyzed the expression data by using a weighted gene co-expression network (WGCNA) and found four biomarkers for two clinic features, respectively. Last, we constructed a DSD-based DNA computing system for cancer analysis. The inputs of the system are these identified biomarkers; the outputs are the fluorescent signals that represent their corresponding traits. The experiment and simulation results demonstrated the reliability of the DNA computing system. This DSD simulation system is lab-free but clinically meaningful. We expect this innovative method to be useful for rapid and accurate cancer diagnosis.

## Introduction

There are two primary methods involved in cancer diagnosis. One is the identification of key biomarkers in gene chips by using bioinformatical methods, such as differential expressed genes (DEGs) array, clinical traits analysis (Xu et al., 2000; Ryu et al., 2002). The other involves clinical methods, for example, tumor site angiography, blood investigation, extracellular vesicle test, etc. (Maton et al., 1987; Masilamani et al., 2004; Verma et al., 2015). Using clinical procedures to diagnose diseases is reliable, but laborious and time-consuming. With the help of bioinformatics technology, we can analyze vast amounts of data and statistically identify critical biomarkers (Li et al., 2003; Abeel et al., 2010). Still, those biomarkers, hub genes, and proteins require verification. Therefore, a clinically reliable, simple, and efficient method is needed.

Recently, microRNAs (miRNAs) have been frequently employed as cancer-specific biomarkers (Yanaihara et al., 2006; Reddy, 2015). miRNAs are small and highly conserved non-coding RNAs that are endogenously expressed in cells. They functioned as the key regulators for gene expression. miRNAs have been found in a variety of body fluids and their quantity is stable. Therefore, miRNAs are ideal biomarkers for cancer diagnosis (Schwarzenbach et al., 2014).

Researchers have established many miRNA datasets, for example, the Cancer Genome Atlas (TCGA), Gene Expression Omnibus (GEO), International Cancer Genome Consortium (ICGC), etc. To identify the hub-gene in those datasets, bioinformatical methods, for example, the edgeR (Robinson et al., 2010), limma packages (Ritchie et al., 2015) from the Bioconductor project (Anders et al., 2015), provide a complete set of statistical methods for dealing with these data.

DNA as a natural material is biocompatible and programmable (Seeman, 2003; Rothemund, 2006). It can be assembled into arbitrary 2D shapes as well as many 3D structures (Shi et al., 2015; Shi et al., 2016; Ong et al., 2017; Chen et al., 2020a; Chen et al., 2020b; Chen et al., 2022a). DNA nanostructures can function as drug transporters for tumor-specific delivery (Lee et al., 2012; Linko et al., 2015). Recently, DNA based electrochemical biosensors have been introduced as a novelty approach for pathogen detection (Zhang et al., 2018; Kwon et al., 2020). In the field of information science, DNA can be used for computing. Various DNA computing models have been performed (Xu, 2016; Xu et al., 2018; Song and Reif, 2019; Woods et al., 2019). Among them, the DNA Strand Displacement (DSD) computing model is highly efficient. DSD was proposed by Yurke et al. (Turberfield et al., 2003). It involves strand displacement with toehold mediates. Moreover, individual DSD units can be cascaded to form multilayer, multiple inputs and outputs computing systems.

Based on the DSD mechanism, many computational works have been accomplished. Qian et al. designed the seesaw gate and scaled those gates up to a larger digital computing circuit (Qian and Winfree, 2011). John Reif et al. formed renewable time-responsive DNA circuits (Garg et al., 2018). Fan et al. (Wang et al., 2020) implemented digital computing using DNA-based switching circuits. Chen et al. developed the tic-tac-toe game *via* DSD (Chen et al., 2022b). Zhu et al. created a DSD-based DNA encryption system (Zhu et al., 2022). Odd parity checkers (Eshra and El-Sayed, 2014), Sigmoid functions (Salehi et al., 2018), and even larger-scale neural networks (Qian et al., 2011) can be implemented.

**FIGURE 1:**
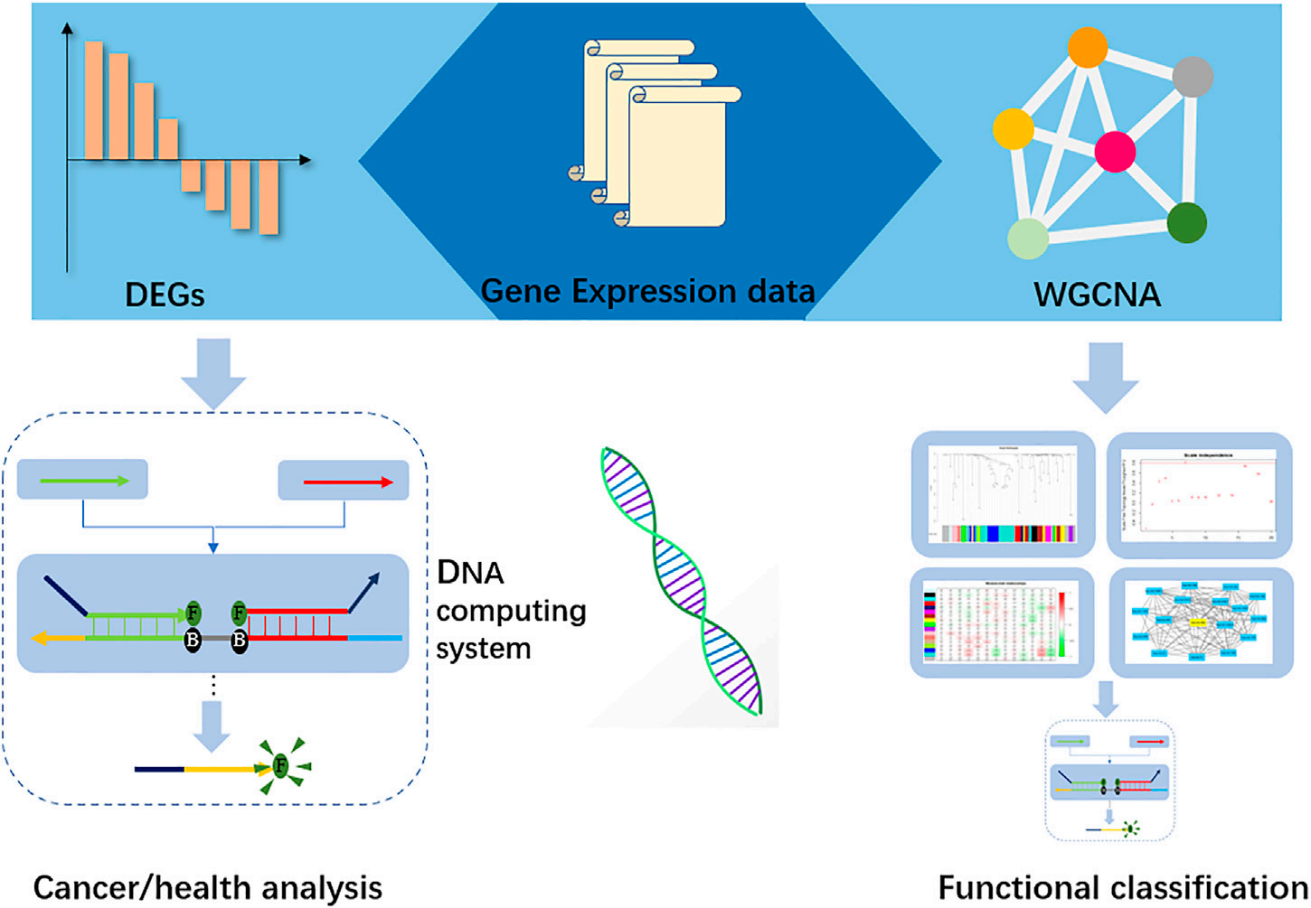
Workflow of the DNA computing system. Gene expression data were used to identify the biomarkers in Differential expression (DEGs), and hub-genes were found by Weight gene co-expression network analysis. Those biomarkers and hub-genes were submitted to the DNA computing system for cancer analysis and functional classification, respectively.

In this research, we constructed a two-layer DNA computing system to analyze gastric cancer and its functional traits. We obtained the miRNA data from ATGC datasets, and then differential analysis methods were applied to seek the distinct miRNA biomarkers. Further, a weighted gene co-expression network (WGCNA) (Langfelder and Horvath, 2008) was established to find the correlated clinical traits and gene sets. The hub genes in those sets were selected as biomarkers for functional classification. In the DNA computing system, those biomarkers were the inputs. We implemented the “winner take all” strategy for cancer analysis. The computing accuracy is up to 80%. Last, we integrated the DSD logic gates and synthesized the microRNAs’ cDNA. These cDNAs were used as inputs. Fluorescence intensity was applied to represent the output signals. Figure 1.

## Materials and methods

The synthetical oligonucleotides used in this experiment were purchased from Sangon Biotech Co., (Shanghai, China) with Ultra-Page purification. Fluorescent modified strands were dissolved in pure water without ions. General strands were dissolved in 1×TE Buffer (40 mM Tris base, 20 mM acetic, 2 mM EDTA, pH = 8.0). All dissolved strands were m Ieasured with a UV 260 nm spectrophotometer and stored at 20°C.

All the gates are assembled by fluorescent molecules and quenched molecules in a 1:1 ratio, acorroding to their design scheme. And then the mixed solution was incubated in a thermal cycle machine, from 50°C to 4°C. The fluorescent signals were measured using ABI QuantStudio Plus (Thermofisher, United States). The mixed gates should be maintained at 4°C before adding the input strands. The fluorescent signal was measured every 10 s, 100 times. The trajectory lines were drawn by Python Echart.

RNA sequence data and corresponding clinic data of gastric cancer were obtained from the ATGC dataset. There are 46 normal cases and 443 cancer cases with miRNA expression quantitative traits. Samples without clinic profiling were deleted (Supplementary Excel S1). Gene expression differential analysis was implemented by “limma” R package (Ritchie et al., 2015). The logarithmic fold changes of UP&DOWN regulated genes (LogFC>1) and *p*-value (*p* < 0.01) were selected to screen out DEGs.

## Results and discussion

### DNA computing for cancer diagnosis

**FIGURE 2:**
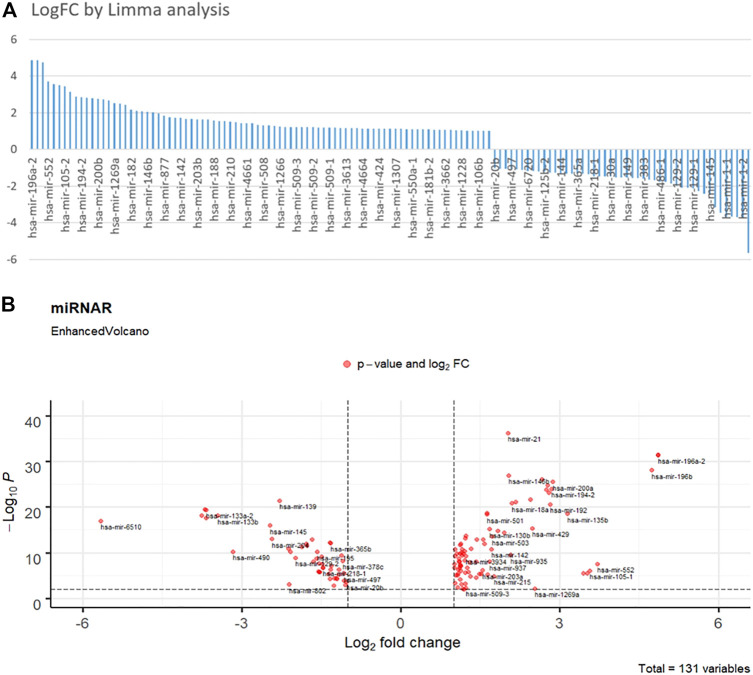
**(A)** Fold change histogram table of the miRNA candidates. **(B)** Enhanced Volcano picture of 131 selected miRNAs. The deep red dots are overlapped miRNAs. Top right and left dots are those significance different miRNAs.

Figure 2 depicts the findings of the limma analysis. We chose the top two up- and down-regulated miRNAs (Figure 2A) as cancer and health input biomarkers, respectively. hsa-mir-196a-1 and hsa-mir-196a-2 are cancer-representative inputs (positive group). The health-represented inputs (negative group) are hsa-mir-6510 and has-mir-1–2. Subsequently, the input sequences of those strands were converted to their corresponding cDNA sequences for DNA computing. Their FPKM (Fragments Per Kilobase Million) value of gene expression data represents their concentration. Additionally, we set the logFC value as the DSD binding rate, that is logFC×k , k is default binding parameter ( k is 0.003nM/s^−1^).

**FIGURE 3:**
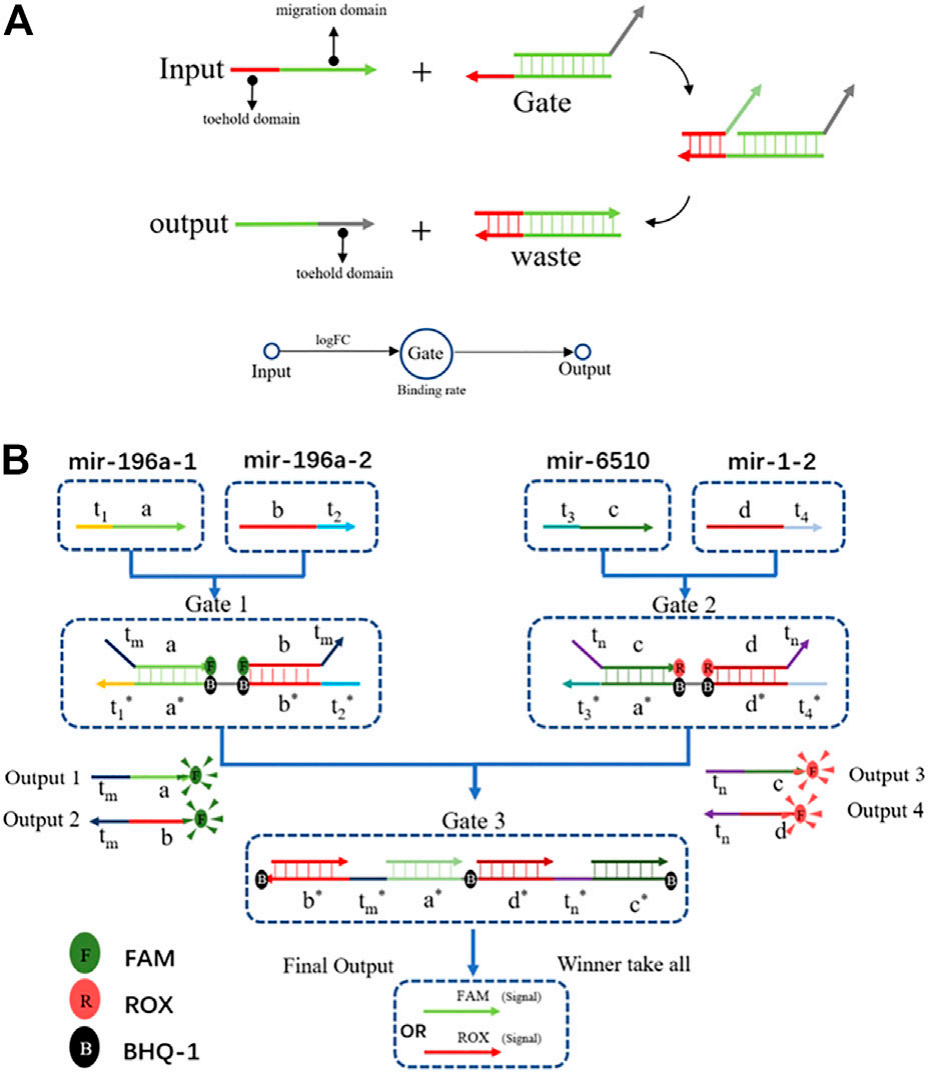
**(A)** The DSD mechanism, the input strand has two domains: the toehold domain and the migration domain. Input strand can displace the output strands with toehold domain mediated. It is a simple one input one output DSD system. **(B)** The DSD computing system. Four inputs one outputs system. Output results were reported by fluorescent dyes.

The DSD mechanism involves two domains: the toehold domain, and the migration domain. The toehold domain will bind to the unpaired area with its corresponding gate, then its migration domain will displace the paired area. Finally, the output strand will be replaced at the gate (Figure 3A). The computing process consists of two steps. First, inputs of the positive and negative groups were added together, respectively. Then, we subtracted the two groups to get the dominant one. In summary, it is a simple winner-take-all strategy with four inputs and a two-layer DNA computing system (Figure 3B).

The output strands were modified with fluorescent dyes, and they were implemented as the report signal. In this system, the toehold binding rate is an important factor that affects the reaction efficiency. The binding rate is a default factor in the DSD simulation system, which is the unit of concentration^−1^ time^−1^ (binding = k, 0.003 nM/s^−1^). LogFC is the value of the average expression of the case group minus the average expression of the control group. It represents the fold change of each target. We directly entered this value into the DSD simulation system, and the binding rate will be processed under the guidance. As a result, the miRNA reaction rate will be limited.

**FIGURE 4:**
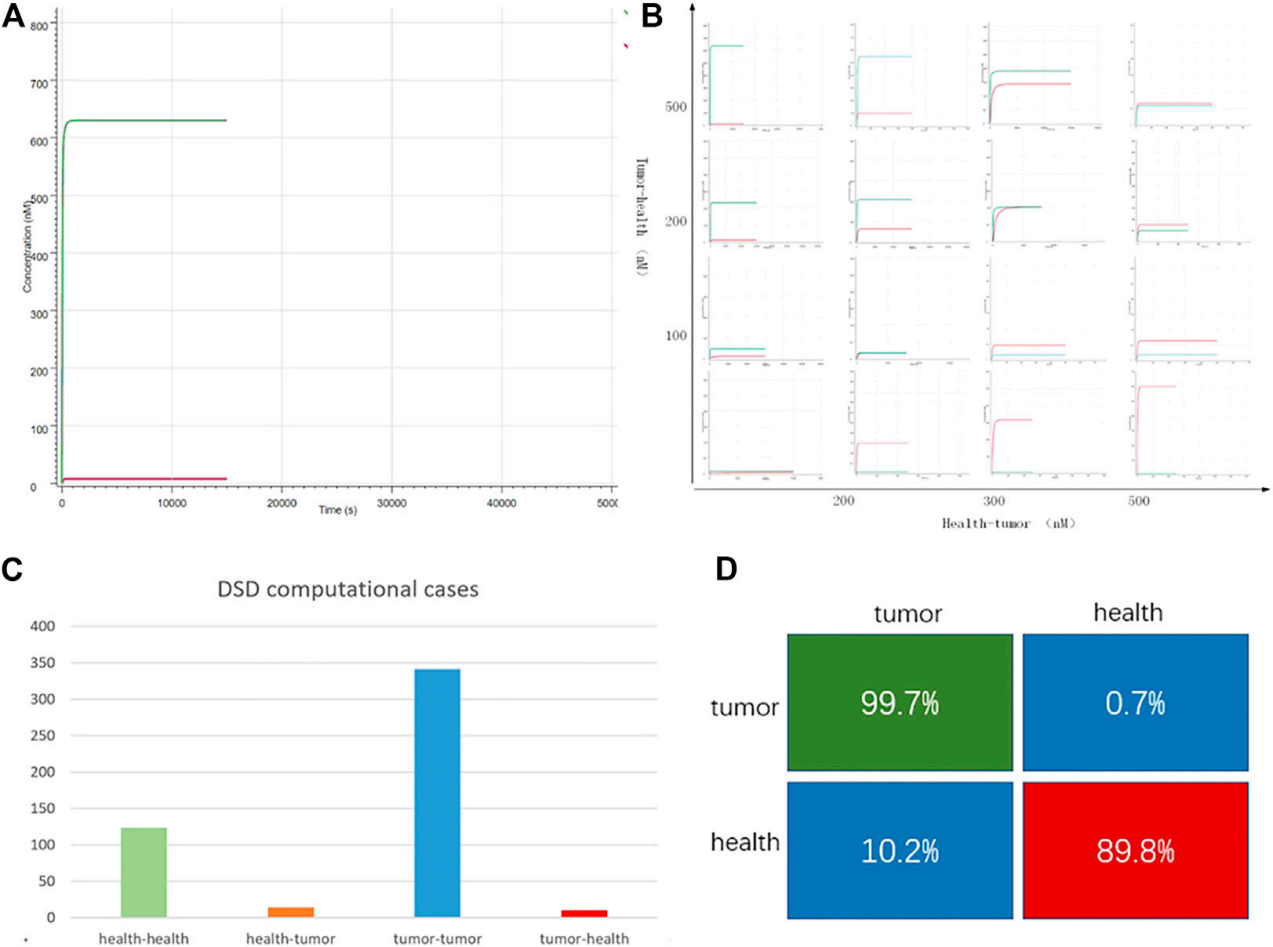
DNA computing results. **(A)** Case “TCGA-3M-AB46-01”: *y*-axis is the output strand’ concentration; the *x*-axis is the reaction time. The green line is the tumor group with FAM fluorescent labels. The red line is the health group with ROX labeled. The green line is higher than the red line, which indicates, in this case, the tumor group predominates. **(B)** 16 typical cases. The whole 490 DSD computing results are attached in Supplementary Material S2. The *x*-axis represents the difference in the concentration of the input DNA strands, the *y*-axis indicates the difference in the fluorescent intensity of the outputs **(C)** DSD simulation results, there are 138 health cases computed as health, 14 health cases computed as tumor. In all the 353 tumor cases, 342 cases were computed as tumor, and 11 cases were computed as health. **(D)** The computational accuracy of tumor cases is 78.7%, while the accuracy of health cases is 92.7%.

The chosen miRNAs served as the inputs for the DNA computing system. We set their FPKM value as the input strands’ concentration. The logFC values were set to the binding rate, that is logFC×k. As illustrated in Figure 3B, mir-196a-1 and mir-196a-2 were the tumor group, when combined with gate1, they generated output strands 1&2. In the tumor-representing group, the output strands were FAM-labeled. In the health-representing group, mir-6510 and mir-1-2 acted with gate2 and produced output strands that were labeled with ROX. Last, all the output strands competed in gate 3. The concentration of gate 3 is the average concentration of mir-6510 and mir-1-2, which is set at 17 nM. Running through gate 3, the winner will take all. Then, we can differentiate between tumor and healthy tissue based on their respective fluorescence signals. The DNA computing results are shown in Figure 4.

Figure 4 shows the DNA computing results. The DSD reactions can be finished in a few minutes, which means the computing system can quickly get the detection results. We noticed that the computational results were related to the tumor and health group’s concentration. The greater the differences between tumor and health, the more distinct the computed results. All the computation results are attached in Supplementary Excel S2. The statistical results are shown in Figures 4C,D, and the confusion matrix shows that DNA computing accuracy is 99.7% for tumor detection and 89.8% for health detection. The total computational accuracy in clinical cases is 94.7%. It should be noted that PFKM values may be subject to measurement errors, which affect the computation precision.

**FIGURE 5:**
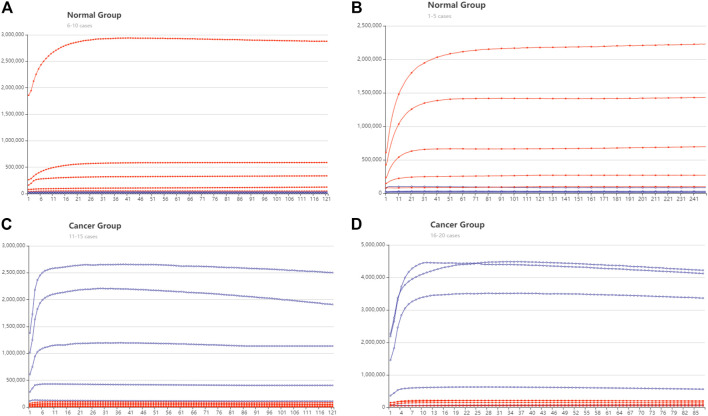
DNA computing with fluorescent signal results. The red lines represent the trajectory of ROX modified signal, and the blue line is the trajectory of the FAM signal. The *y*-axis is Fluorescent intensity, *X* axis is the cycle number, 10 s is one cycle **(A)** The normal group cases 1–5. **(B)** The normal group cases 6–10 **(C)** The cancer group cases 11–15. **(D)** The cancer group cases 16–20.

Following the DSD system, we then validated these four miRNA biomarkers *via* their synthesized cDNAs. We utilized the K-means (K = 10) approach to classify 46 normal cases and 443 cancer cases, each into 10 groups. We believe that these twenty cases are broadly typical. Then, the main case inside each class was selected for validation. The FPKM values of these 20 cases are illustrated in Supplementary Excel S3. We verified these 20 cases by adding the synthesized cDNA based on their FPKM values (TCGA original data), the concentration unit is nM. The cancer-related strands were synthesized with FAM fluorophore modification, and normal-related strands were ROX fluorophore modification. Figure 5 shows the fluorescent results.

There are 16 cases that can be clearly distinguished. The ROX fluorescent is predominant in the health group from cases 1 to 10. whereas in the cancer group, FAM fluorescent is predominant. The DSD computing accuracy is 80%. The remaining four cases are reduced to negligible signals. We assume that this is because the concentration of target cDNAs in the four cases is too low to be identified. Because their concentration is less than the threshold gate 3. Actual fluorescent signal data are included in the Supplementary Excel S2–S5.

Further, we constructed a weighted gene co-expression network (WGCNA) and selected some biomarkers for the positive and negative correlated groups. Then we used those biomarkers as inputs of the DNA computing system for functional classification.

### DNA computing with weighted gene co-expression network-based clinical classification

WGCNA is a whole-inspection method to analyze genes and traits. WGCNA treats genes as a whole set that are closely connected to a certain clinical trait. In the set, genes were depicted as points, and gene-to-gene relationships were depicted as edges. The gene-to-gene correlation was calculated by its expression value. Therefore, genes were connected to form a scale-free network. Then, the clustering method was applied; typical genes were clustered as a set (gene module). Last, a module-to-clinical trait relationship was established, and those modules were depicted as positive or negative correlates to one specific clinical trait.

**FIGURE 6:**
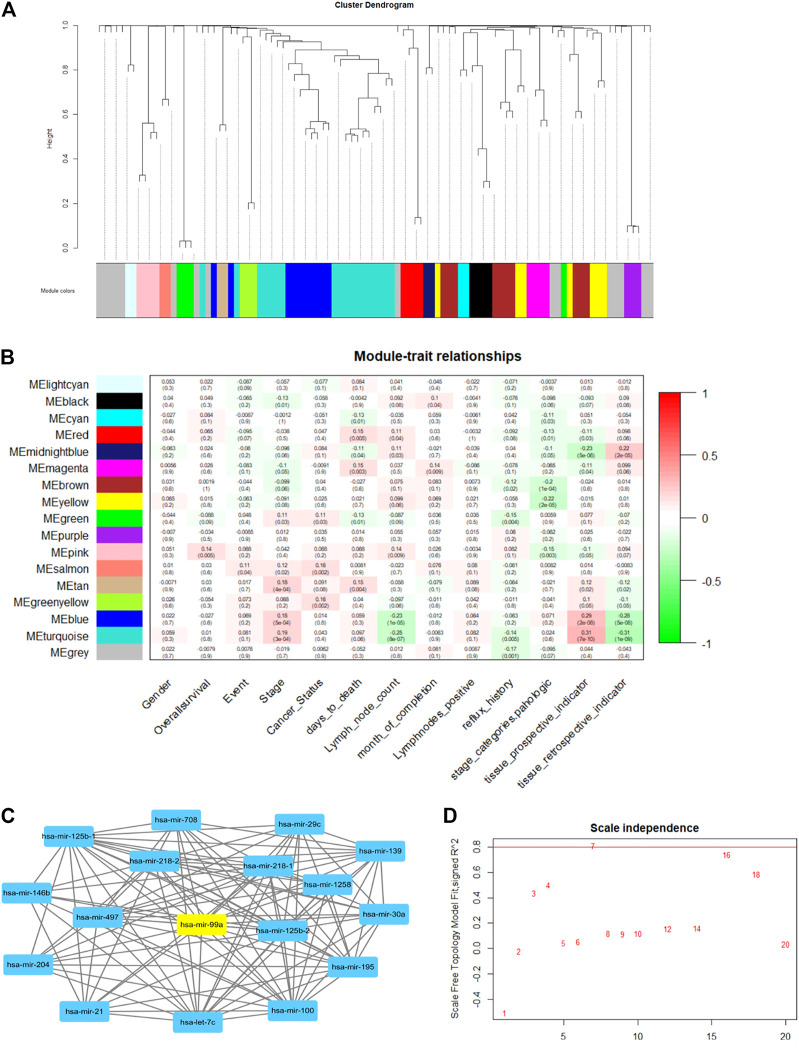
The example of WGCNA network analysis. **(A)** The gene cluster dendrogram. Genes are clustered into 17 modules, which are illustrated in different colors. **(B)** Module-trait relationships: there are 13 clinic traits. Module and trait relationships are illustrated in red and green. The red color shows that they are positively related, and the green color means they are negatively related. **(C)** Example of a gene topology graph in the turquoise module. The hub gene in this module (has-mir-99a) is highlighted in yellow. **(D)** gene-gene scale independence and module fit relationship. The red line is the suitable threshold for module fit. We chose power = 7 as the best factor, as a higher power value will make the gene topology graph sparse.

In this work, we used the WGCNA R package (Eshra and El-Sayed, 2014) to perform the network and construct a module-trait matrix. First, the gene-to-gene relationship was calculated by the covariance function. Then, the power function was used to exclude weak connections, which is called the soft-thresholding power. It is a key factor that decides the mean connectivity of the genes. Typically, the soft-thresholding power ranges from 1 to 20. A suitable power value makes the topology model fit index over 0.8 (model scale between 0 and 1). The index and power results are illustrated in Figure 6D. Second, genes were clustered using hierarchical clustering, and gene modules are illustrated in different colors. The cluster dendrogram is depicted in Figure 6A. Last, the modules-trait relationship was established by using Pearson correlation, as shown in Figure 6B.

Figure 6 shows the WGCNA analysis results. After calculating power and clustering, genes were divided into 17 modules. The modules-trait relationship is illustrated in Figure 6B. The value in each panel is the module-trait relationship value. We found that the MEturquoise group and MEmidnightblue group had a strong positive and negative correlation with the tissue prospective indicator, respectively. (Tissue retrospective indicator and the tissue prospective are two correlated traits). Therefore, MEturquoise and MEmidnightblue can represent those two clinical traits. Furthermore, we discovered that MEturquoise and MEpink have a weak correlation with lymph node counts. We chose the hub-gene in each module as the biomarker for DNA computing (the hub-gene in the same module changes when it correlates to different clinical traits). Figure 6C shows the hub-gene (hsa-mir-99a) in turquoise when correlated to lymph node count. The hub gene was calculated by adding all the weight values of connective nodes.

**FIGURE 7:**
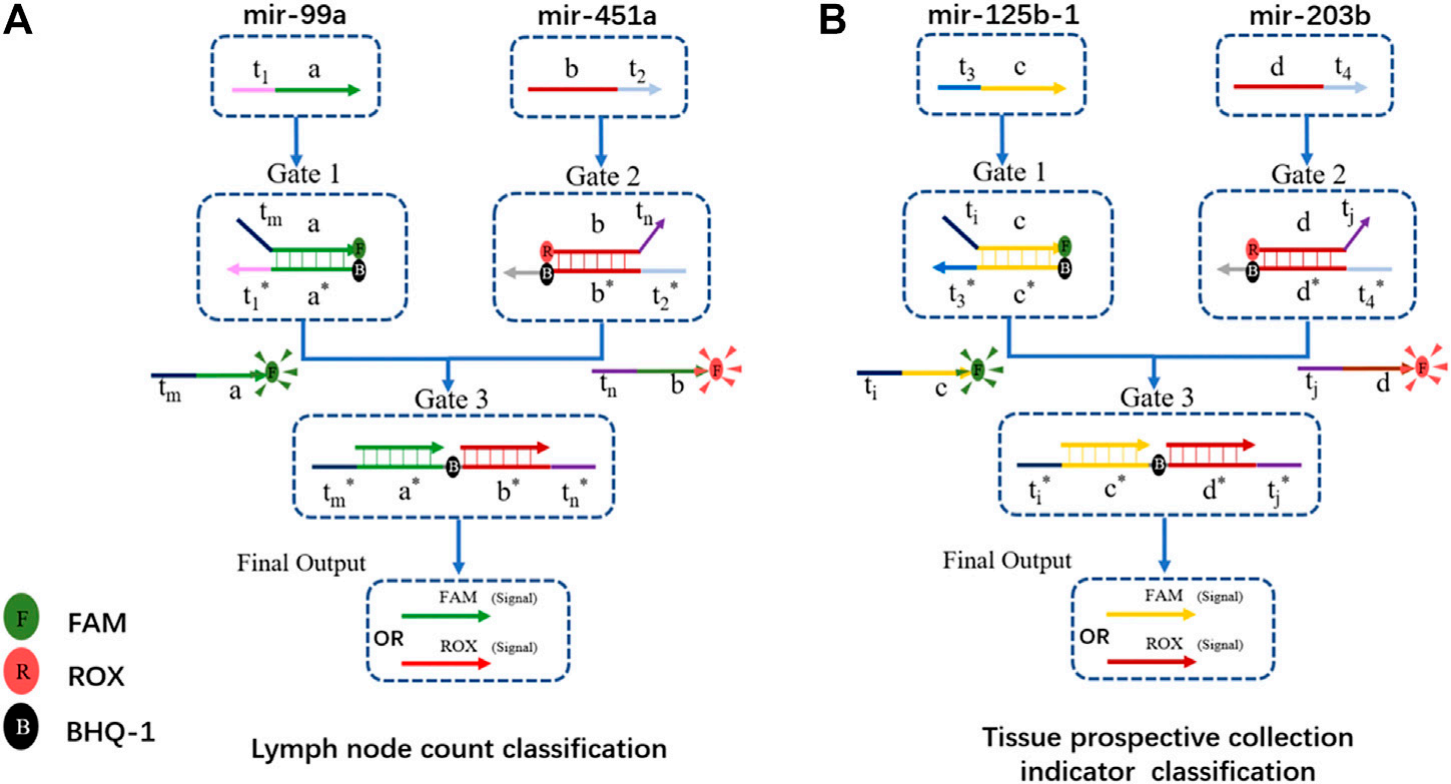
DSD-based DNA computing system for functional classification. **(A)** Gene biomarkers mir-99a and mir-451a for lymph node examined count classification. FAM and ROX were attached to the final output strand. FAM/ROX dominates the high/low expression of lymph node counts. **(B)** Gene biomarker mir-125b-1 and mir-203b for tissue prospective collection indicator value classification. FAM/ROX dominates the high/low expression of this trait.

We chose the lymph node count, tissue prospective collective indicator as the two clinical traits for functional classification. The hub gene with positive and negative ties is chosen as the input. Therefore, hsa-mir-99a in MEturquoise and hsa-mir-451a in MEpink were the inputs for lymph node count classification. Has-mir-125b-1 in MEturquoise and has-mir-203b in MEmidnightblue are two inputs for the tissue collection indicator. The DNA computing systems are shown in Figure 7.

**FIGURE 8:**
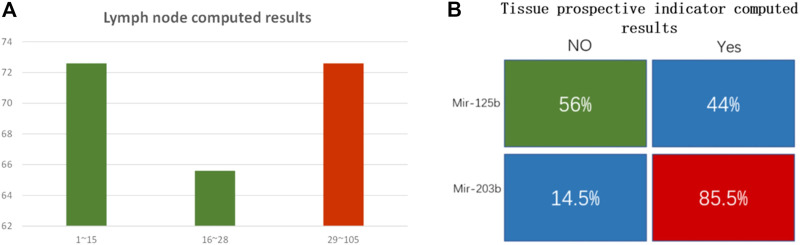
**(A)** Lymph node number computed results. The *y*-axis is computational accuracy. The *x*-axis is the node number. Green bars show the mir-99a FAM modified group. The red bar shows the mir-451a ROX modified group. When the lymph node number is under 15, FAM dominates, and the computed accuracy is 72.6%; when the node number is between 16 and 28, FAM dominated, and the computing accuracy is 65.6%; when the node number is between 29 and 105, ROX strand dominated, the DNA computing accuracy is 72.7%. **(B)** Tissue prospective collective indicator computed results. The indicator “Yes” or “No” was transformed to 1 or 0. Mir-125b correlates to the 0 indicator, and the computed accuracy is 56%; mir-203b correlates to 1 indicator, and DNA computed accuracy is 85.5%.

The structure of the DNA computing system for those two clinical traits is the same, except for the Gates sequences, which were correspondingly modified. We chose hub-gene in each module as the inputs. These positive and negative related biomarkers reacted with the two-layer DSD-based system, and produced the final strands that are labeled with either FAM or ROX signal. The final output correlates with the expression of its clinical trait. If the FAM signal predominates in the lymph node trait, it implies fewer lymph nodes. If ROX is dominant, the situation is the opposite. In the tissue collection indicator trait, FAM/ROX correlates to the high/low expression of tissue number, respectively. Figure 8 shows the DNA computing results (statistics results are included in Supplementary Excel S4).

In clinical analysis, researchers usually set node number 15 as the survival threshold for prognostic criteria (Liang et al., 2017; Xia et al., 2019). Patients with node numbers higher than 30 have a poor survival rate. In the lymph node examined group, we divided the node count into 3 parts, from low to high. There are 190 cases in part one (node number lower than 15), 96 cases in part two (node number 16–28), and 106 cases in part three (node number 29–105). DNA computing results showed that mir-99a dominated in the lower node number parts. The accuracy was 72.6% and 65.6% in part one and part two, respectively. The average accuracy for mir-99a was 70% (201 correctly computed cases out of all 286 cases). Mir-451a dominated in the higher node part, DNA computing accuracy is 72.7%. The DNA computing accuracy for this trait is not high enough. We suppose the reason lies in the coefficient value. The coefficient *p* value for miRNA biomarkers and lymph node trait is 0.25, which is not high. It indicates that the connection between miRNA and this trait is weak.

The tissue prospective collection indicator has only two values: “Yes” and “No.” We found mir-125b has no clear association with tissue indicator, as the computed result was low (56%). However, that is reasonable. “No indicator” means there are no obvious carcinogenic factors connected with this trait. While in the “Yes” group, mir-203b is highly correlated, the DNA computing accuracy was 85.5%. The statistical results are included in the Supplementary Material.

**FIGURE 9:**
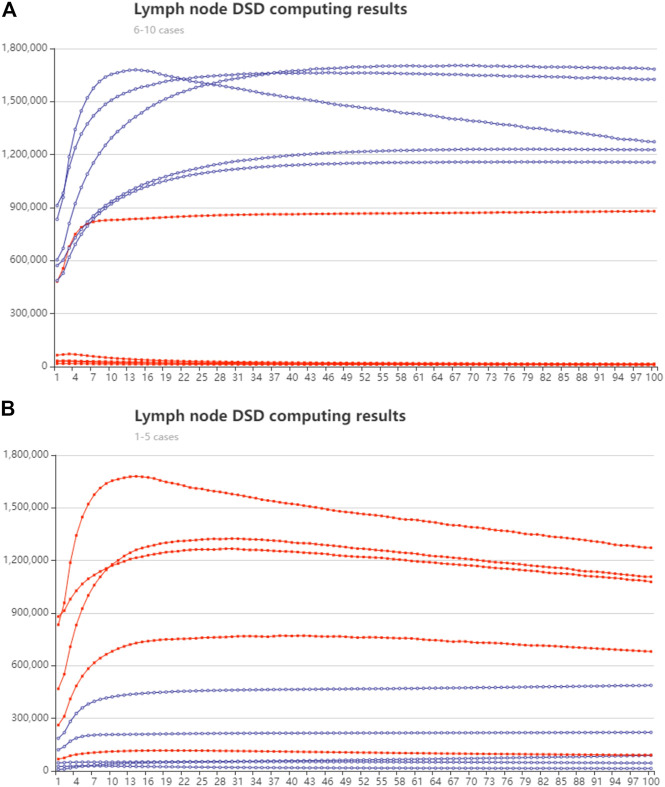
Lymph node number classification with DSD computed results. The red lines represent the trajectory of the ROX modified signal, and the blue line is the trajectory of FAM signal. The *y*-axis is fluorescent intensity. *X* axis is the cycle number, 10-s pre-time **(A)** Group one, lymph node fewer than 28, cases 6–10 Biomarker mir-99a predominates. **(B)** Group two, lymph node greater than 28, cases 1–5. Mir-451a predominates.

We randomly chose five cases in lymph node samples that were less than or over 28, respectively. The synthesized cDNA strands were added to the DSD system. The concentration of the input strands follows their expression number in nM units. The strands in group one (less than 28 nodes) were modified with FAM fluorophore, and group two (over 28 nodes) were modified with ROX fluorophore. The DSD computing results are illustrated in Figure 9. The real-time fluorescence data is illustrated in Supplementary Excel S5. Excel instructions are added.

The DNA computing outcomes were not perfectly associated with simulation or statistical outcomes. We found some turbulence and fluctuations in the fluorescent signals. We think deviation could be generated in manual operations. Leakage may accrue during the DNA strand’s reaction process. The red and blue lines show the accurate reaction process. Therefore, we did not amend these deviations. Additionally, the red and blue lines show the correct computing results (high/low signal).

DNA computing for tissue prospective accuracy is 85%. While the lymph node statistical classification accuracy is 70%–80%. We believe that the cause resides in model-trait connections. The hub gene in those two modules did not have a strong relationship with its clinic trait, which hampered the computing result. But we can diagnose cancer and classify cancer traits using DNA computing.

## Conclusion

In conclusion, we proposed a novel method that uses DNA computing to analyze cancer traits. This DSD-based DNA computing system used biomarkers (or hub-genes in WGCNA) as the inputs. The concentration of input strands is their expression value. The LogFC value of each input strand was set to the binding rate. The DNA computing accuracy for cancer health identification is 94.7%. The computing accuracy of these two clinical traits is 70% and 85.5%, respectively.

Furthermore, we think selecting suitable biomarkers as the inputs can improve the accuracy of this system. WGCNA is a powerful tool to identify the biomarker (the hub-gene in each module). However, the hub gene might deviate from the clinical trait (when the *p*-value is high). Therefore, we are seeking more efficient bioinformatics methods to locate suitable biomarkers. Further, we will attempt to construct a multi-layer, serial/parallel combined DNA computing system for complex cancer trait examination.

## Data Availability

The original contributions presented in the study are included in the article/Supplementary Material, further inquiries can be directed to the corresponding authors.
